# Berries and Leaves of *Actinidia kolomikta* (Rupr. & Maxim.) Maxim.: A Source of Phenolic Compounds

**DOI:** 10.3390/plants11020147

**Published:** 2022-01-06

**Authors:** Laima Česonienė, Paulina Štreimikytė, Mindaugas Liaudanskas, Vaidotas Žvikas, Pranas Viškelis, Jonas Viškelis, Remigijus Daubaras

**Affiliations:** 1 Botanical Garden, Vytautas Magnus University, Z.E. Zilibero 6, LT-46324 Kaunas, Lithuania; remigijus.daubaras@vdu.lt; 2 Lithuanian Research Centre for Agriculture and Forestry, Institute of Horticulture, LT-54333 Babtai, Lithuania; pranas.viskelis@lammc.lt (P.V.); jonas.viskelis@lammc.lt (J.V.); 3 Department of Pharmacognosy, Faculty of Pharmacy, Lithuanian University of Health Sciences, LT-50166 Kaunas, Lithuania; mindaugas.liaudanskas@lsmuni.lt; 4 Institute of Pharmaceutical Technologies, Faculty of Pharmacy, Lithuanian University of Health Sciences, LT-50166 Kaunas, Lithuania; vaidotas.zvikas@lsmuni.lt

**Keywords:** *Actinidia kolomikta*, antioxidant activity, berries, cultivar, phenolic compounds, UHPLC-ESI-MS/MS

## Abstract

Berries of *Actinidia kolomikta* (*A. kolomikta*) are known for high ascorbic acid content, but the diversity of phenolic compounds has been little studied. The present research aimed to investigate phenolic compounds and antioxidant activity in berries and leaves of twelve *A. kolomikta* cultivars. The UHPLC-ESI-MS/MS technique was used to determine differences among cultivars in the quantitative composition of individual phenolic compounds. Antioxidant activity was determined by DPPH• free radical scavenging and CUPRAC methods. In the present study, 13 phenolic compounds were detected in berries, whereas leaves contained 17 phenolic compounds. Flavonols were the primary class found in both berries and leaves; other identified phenolic compounds were flavan-3-ols, flavones and, phenolic acids; and dihydrochalcone phloridzin was identified in the leaves. The amount and variety of phenolic compounds in berries and leaves and antioxidant activity were found to be cultivar-dependent. The highest total content of phenolic compounds was found in the leaves of the cultivar ‘Aromatnaja’ and in the berries of the cultivar ‘VIR-2’. Results of this study have confirmed that berries and leaves of *A. kolomikta* could be a valuable raw material for both food and pharmaceutical industries.

## 1. Introduction

Quantitative and qualitative research on the various biochemical compounds derived from fruits, including berries, is becoming the basis for innovative products in the pharmaceutical, cosmetic, and food industries. Fruits and their products possess a high concentration of phenolic compounds (flavonols, phenolic acids, flavan-3-ols, flavones, and others); and the antioxidant activity of phenolic compounds in vitro and in vivo has been proven [1,2]. Individual phenolic compounds or various combinations can have a substantial effect on human health. Protection against cardiovascular diseases, inflammation, and cancer have been confirmed [3,4,5].

Studies of berries of various *Actinidia* Lindl. species have shown higher levels of phenolic compounds than apples, grapefruits, lemons, or peaches [6,7]. A review of studies on phenolic compounds showed that berries of different *Actinidia* species were characterized by both quantitative and qualitative diversity of these compounds. Biologically active compounds of species *A. deliciosa* (A.Chev.) C.F.Liang & A.R.Ferguson and *A. chinensis* Planch. have been studied in more detail using state-of-the-art equipment and modern methods [8,9,10]. In recent years, more attention has been paid to *A. arguta* (Siebold & Zucc.) Planch. ex Miq., *A. arguta* var. *purpurea* (Rehder) C.F.Liang ex Q.Q.Chang and their hybrid cultivars [11,12,13]. Zuo et al. found that phenolic compounds were the major contributors to the total antioxidant capacity of *Actinidia* berries [14].

Phenolic compounds are usually concentrated in the skin of *A. arguta* and *A. deliciosa* berries. The phenolic content in the berry skin can be up to ten times greater than that of the berry pulp [15]. *A. arguta* leaves were also investigated for biochemical compounds and, antioxidant and antimicrobial activity [16]. The results of this study confirmed the presence of phenolic compounds from flavan-3-ols, flavonols, and hydroxycinnamic acid classes and verified the antimicrobial activity of leaf extracts. Other authors ascertained that berries of winter-hardy *Actinidia* species accumulated significantly higher amounts of phenolic compounds than the world’s most widely grown cultivars of *A. deliciosa* and *A. chinensis* [12,15,17]. Eventually, phenolic compounds of *A. deliciosa*, *A. chinensis* and *A. arguta* berries could be highly appreciated for the health-promoting benefits of dietary consumption [17,18,19].

The quantitative and qualitative composition of phenolic compounds significantly influences *Actinidia* berries’ quality and taste properties [20,21,22]. The results of different studies have corroborated that phenolic compounds play an essential role in plants’ defensive reactions, i.e., environmental stress can raise the accumulation of phenolic compounds, including phenolic acids [23,24,25,26]. *Actinidia kolomikta* (Rupr. & Maxim.) Maxim. is the most cold-hardy species and extends north to 52°40′ N, further north than any other *Actinidia* species [27]. Interestingly, *A. kolomikta* cultivars and clones are cultivated because of their exceptional resistance to pests and diseases in Lithuania. So far, this dioecious species has been extensively investigated, and new cultivars have been selected in Russia, Poland, and Lithuania. *A. kolomikta* was commonly cultivated in the temperate zone of Europe due to its winter hardiness and berry harvest. The cultivation of *A. kolomikta* cultivars has so far been mainly of interest to amateur gardeners who have used berries for their own purposes. On the other hand, long-term investigations on *A. kolomikta* have confirmed pest resistance, which supports cultivation in organic plantations [28]. This study aimed to detect the diversity of phenolic compounds and antioxidant activity in berries and leaves of *A. kolomikta* and determine variation among cultivars.

Few studies have been published on the biochemical composition of *A. kolomikta* berries. Biologically active substances, namely vitamin C, total phenolic, and total flavonoid compounds, have been presented in some studies by different authors [14,17,29]. Nevertheless, *A. kolomikta* has not been properly assessed for its composition of biologically active compounds. Investigations on secondary metabolites in berries and leaves, including phenolic compounds, are relevant because of the growing interest in their multiple biological effects.

## 2. Results and Discussion

### 2.1. Determination of Phenolic Compounds Using UHPLC-ESI-MS/MS

The results of this study confirmed some quantitative and qualitative differences in phenolic compounds between berries and leaves of *A. kolomikta*. A total of 13 phenolic compounds in the berries and 17 phenolic compounds in the leaves were identified. In the berries and leaves, the major flavonols identified were kaempherol, kaempherol-3-*O*-glucoside, quercetin, quercitrin, isorhamnetin, and rutin (Table 1 and Table 2).

Flavonols isorhamnetin-3-*O*-rutinoside and galangin were identified only in leaves of the cultivars ‘Anykšta’, ‘Sentiabrskaja’, and ‘Aromatnaja’, respectively. Phloridzin (dihydrochalcone) was found in leaves of the cultivar ‘VIR-1’ (Table 1). The total content of flavonols ranged from 727.96 µg/g to 2128.08 µg/g in the leaves and from 157.21 µg/g to 589.01 µg/g in the berries of *A. kolomikta* cultivars (Table 3). Flavon-3-ols (+)-catechin, (−)-epicatechin, and procyanidin C1 represented the second most abundant class of phenolic compounds; but the total amount of flavon-3-ols was the most extensive, measuring on average 36.4% in the berries and 61.6% in the leaves of the total phenolics amount. Three compounds belonging to the hydroxycinnamic acids class were found in the both berries and leaves, i.e., chlorogenic, neochlorogenic, and caffeic acids. High amounts of chlorogenic and neochlorogenic acids were detected in the leaves of all cultivars. Chlorogenic acid was notably predominant in the berries, while caffeic acid was found only in the cultivar ‘Lankė’, and the berries of the cultivar ‘VIR-2’ were distinguished by neochlorogenic acid. Other authors have reported that neochlorogenic acid was the major phenolic acid in berries of *A. arguta* except for ‘Jumbo’, in which chlorogenic acid dominated [5]. For flavone class, acacetin was found in the leaves of some cultivars but this compound was not present in the berries at all. Small amounts of apigenin have been found in both the berries and leaves of many cultivars. It must be acknowledged that the total amount of flavones was insignificant compared to other classes of phenolic compounds and ranged from 0.28 µg/g to 8.9 µg/g and from 0.02 µg/g to 0.09 µg/g in the leaves and berries, respectively. As in our study, the total amount of flavan-3-ols was largest in berries of five cultivars of *A. arguta* cultivars and in two crosses of *A. arguta* × *A. purpurea* [5].

Investigations of phenolic compounds in the berries and leaves of twelve *A. kolomikta* cultivars confirmed that their quantitative and qualitative composition depends on the genotype. In our study, the largest amount of total phenolic compounds was detected in the cultivar ‘VIR-2’ berries (Table 3). The highest concentration of flavan-3-ols including procyanidin C1 was found in berries of the cultivar ‘VIR-2’, 1468.27 µg/g and 1236.66 µg/g, respectively and the lowest in ‘Matovaja’, 436.11 µg/g and 316.04, respectively.

Leaves of the cultivar ‘Aromatnaja ‘were characterized by exceptionally high content of phenolic compounds (7679.72 µg/g), of which 63.7% were flavan-3-ols. The amounts of phenolic acids in the leaves of different cultivars varied greatly because the leaves of the cultivar ‘Aromatnaja’ accumulated 1193.61 µg/g of phenolic acids whereas in the leaves of the cultivar ‘Landė’ 52.97 µg/g of phenolic acids were present. Other authors have indicated that the amount of phenolic compounds in *A. arguta* berries was also significantly cultivar dependent [18]. Examination of *A. arguta* cultivars ‘Ananasnaja’, ‘Geneva’, ‘Weiki’, and ‘Issai’ showed that the berries of the cultivar ‘Ananasnaja’ accumulated the most phenolic compounds (6679.18 mg/100 g DW) [5].

To the best of our knowledge, little research has been carried out on the biochemical composition of *Actinidia* spp. leaves. Almeida et al. found that HPLC analysis revealed the highest amounts of phenolic acids (hydroxycinnamic acid derivatives) and flavonoids (flavan-3-ol and flavonols derivatives) in the leaves of *A. arguta* [16]. In our study, the leaves of *A. kolomikta* cultivars were characterized by significant amounts of phenolic compounds. High amounts of phenolic compounds in the leaves of *A. arguta* have been described by Marangi et al. [30]. Interestingly, young apical leaves were identified as having the highest content of total phenolic compounds and flavonoids, and a continuous decrease in total phenolic content with leaf maturity was detected in eight accessions of *A. arguta* [31].

The predominant phenolic compounds in *A. deliciosa* berries were flavonoids, tannins, and flavonols [29,32]. Zuo et al. summarized the results of *A. chinensis*, *A. deliciosa*, *A. arguta*, and *A. kolomikta* biochemical investigations and confirmed that the total content of phenolic compounds was the highest in the berries of *A. kolomikta* genotypes whereas, the amounts of flavonoids found in *A. kolomikta* and *A. arguta* did not differ significantly [14]. Comparison of different studies on the qualitative and quantitative variety of phenolic compounds has some limitations due to variation in the preparation of extracts and methods used. The classes of phenolic compounds found in the berries and leaves of *A. kolomikta* cultivars were essentially consistent with the data of other authors evaluating the phenolic compounds in different *A. arguta* genotypes [16,29].

In our study, trends of the accumulation of various valuable health-promoting phenolic compounds were similar in both the leaves and berries. Investigations have shown that the highest levels of phenolic compounds in *A. kolomikta* leaves and berries were found for procyanidin C1. As Neyestany reported, this compound was found to be a potent inhibitor of lipid peroxidation [9]. The evaluation of the impact of quercetin on oxidative stress and vascular function confirmed quercetin’s effects on blood [33]. In vitro and in vivo protection of cells from inflammatory injury has been reported for kaempherol–3-*O*-glucoside [34]. Small amounts of the flavonol galangin, and dihydrochalcone phloridzin were found in the leaves of the cultivars ‘Aromatnaja’ and ‘VIR-1’, nevertheless, their biological activity is worthy of attention and further research. In vitro studies have shown that galangin regulates glucose levels, and the effect of phloridzin on human health, especially diabetes, was also substantiated [34,35,36].

### 2.2. Determination of Antioxidant Activity and Correlation with TPC

**Figure 1:**
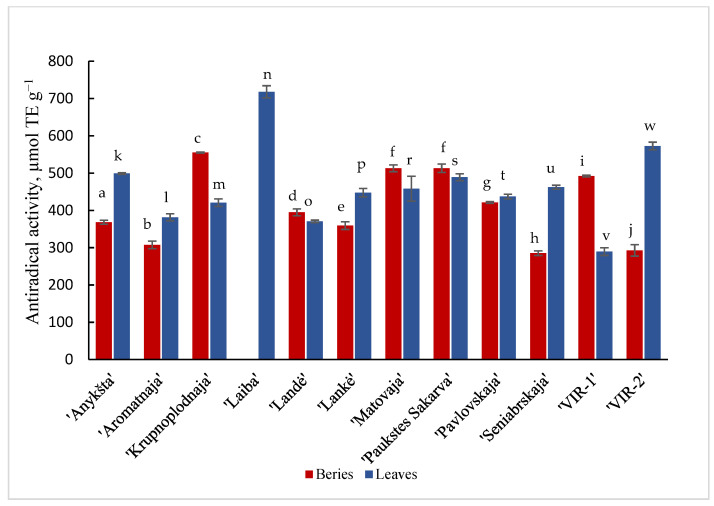
DPPH• antiradical activity of berries and leaves of different cultivars of *A. kolomikta*. Data were expressed as mean ± SD. Different letters above columns means significant difference between berries or leaves according to Duncan’s least significant difference (LSD) procedure at 1% significance level.

**Figure 2:**
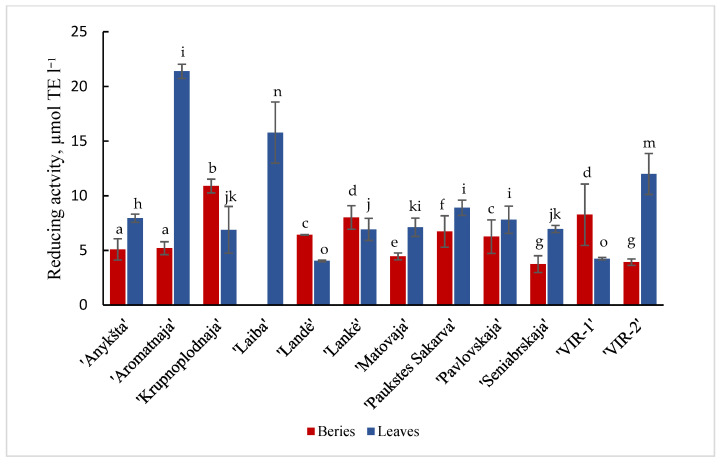
CUPRAC reducing activity of berries and leaves of different cultivars of *A. kolomikta.* Data were expressed as mean ± SD. Different letters above columns means significant difference between berries or leaves according to Duncan’s least significant difference (LSD) procedure at 1% significance level.

Results are shown in Figure 1 and Figure 2 of the antioxidant activity of different cultivars of *A. kolomikta*. Specifically, berries and leaves were taken to measure DPPH and CUPRAC antioxidant activity. Significant variation was found in the antiradical activity of the studied cultivars and the values in berries and leaves varied from 285.1 to 555.21 μmol/TE g^−1^ and from 370.7 to 499.1 μmol/TE g^−1^ (DPPH assay), respectively. These results confirm that leaves of the cultivar ‘Laiba’ and berries of the cultivar ‘Krupnoplodnaja’ had exceptionally high antiradical activity (Figure 1). The CUPRAC method determined the highest reducing activity for ‘Aromatnaja’ leaves and ‘Krupnoplodnaja’ berries. The value of the reducing activity varied from 5.09 to 10.89 μmol/TE g^−1^ in the berries and from 4.04 to 21.38 μmol/TE g^−1^ in the leaves of different cultivars as determined by the CUPRAC assay (Figure 2).

As Wang et al. reported, the antioxidant potential of the commercial cultivars of *A. chinensis* and *A. deliciosa* was strongly affected by the cultivars, and the berries of *A. kolomikta* cultivars differed significantly in antioxidant activity [17]. This could be explained by the fact that previous studies have also confirmed exceptionally high levels of another powerful antioxidant, ascorbic acid, which is also characterized by high antioxidant potential [28].

Studies in various fruits have shown that phenolic compounds may affect antioxidant activity; and berries of *Actinidia* have been shown to have strong antioxidant potential compared to blueberries and cranberries. The accumulation of phenolic compounds depends on various ecological conditions and cultivation technologies [37].

**Figure 3:**
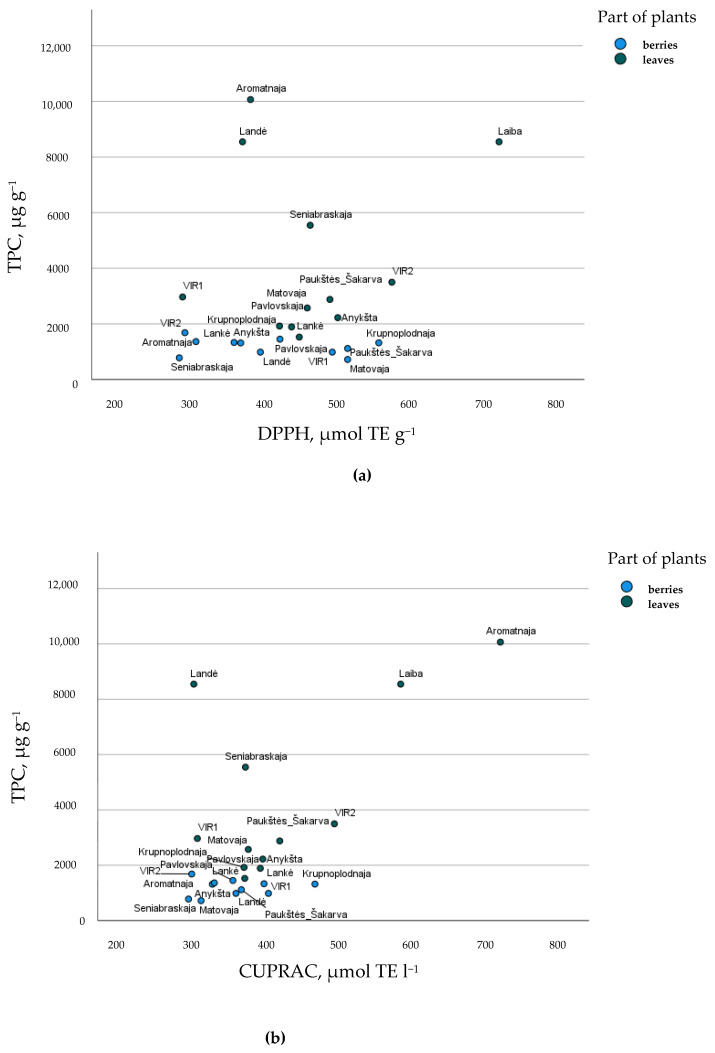
Cultivar’s arrangement describing (**a**) total phenolic compounds (TPC, µg g^−1^) and DPPH, µmol TE g^−1^ antiradical activity and (**b**) total phenolic compounds (TPC, µg g^−1^) and CUPRAC, µmol TE g^−1^ reducing activity of *A. kolomikta* berries and leaves.

Figure 3 provides a graphical representation of the cultivars berries and leaves, according to TPC quantity and antioxidant activity. Some cultivars were shown for the uniqueness of their leaves—‘Laiba’ for DPPH antiradical activity and ‘Aromatnaja’ for CUPRAC reducing activity. This study ascertained that the berries and leaves of different cultivars of *A. kolomikta* accumulated a variety of phenolic compounds with high antioxidant activity from 720.64 µg/g to 1684.48 µg/g and from 1420.44 to 76,679.72, respectively. These results strongly support possible applications for pharmaceutical, nutraceutical, and food industries, and green synthesis for nanoparticles, for example, as a capping and reducing agent in nanoparticle synthesis [38]. 

## 3. Materials and Methods

### 3.1. Plant Material

Berries and leaves were collected in the *Actinidia* spp. field collection of the Botanical Garden of Vytautas Magnus University. This collection was established during the implementation of the Lithuanian State Plant Genetic Resources Research Program in 1998–2018. The collection is located in the Central region of Lithuania, 76 m above sea level. The growing season lasts for approximately 180 days in this region. The species *A. kolomikta.* i.e., five cultivars of Lithuanian origin and seven cultivars of Russian origin were selected for investigation (Table 4).

Approximately 250–300 g of berry samples were prepared per cultivar. Berries were harvested randomly from six plants at the stage of technical maturity (berries had begun to ripen and soften) and then mixed. The time of sample collection depended on the cultivar properties and continued from the last week of July to the first week of August. Berries of the ‘Laiba’ variety were not evaluated due to their insufficient quantity. All samples of leaves were collected in July. Approximately 200 g of fully developed leaves per cultivar were collected from mixed or vegetative shoots, excluding the four lower leaves at the base of the shoot and the five to six upper leaves.

### 3.2. Chemicals

All the solvents, reagents, and standards used were of analytical grade. The following substances were used in the study: ethanol 96% (*v*/*v*) (SC “Vilniaus degtinė”, Vilnius, Lithuania), formic acid, acetonitrile, (−)-epicatechin, (+)-catechin, procyanidin C1, isorhamnetin, acacetin, apigenin, caffeic acid, chlorogenic acid, neochlorogenic acid, phloridzin, kaempherol, kaempherol-3-O-glucoside, quercetin, galangin, quercitrin, rutin, isorhamnetin-3-O-rutinoside, sodium carbonate, gallic acid monohydrate, DPPH (2,2-diphenyl-1-picrylhydrazyl), neocuproine, ammonium acetate, copper (II) chloride dihydrate, trolox ((±)-6-hydroxy-2,5,7,8-tetramethylchromano-2-carboxylic acid) (Sigma-Aldrich, Steinheim, Germany). During the study, we used purified de-ionized water prepared with the “Milli–Q^®^” (“Millipore”, Bedford, MA, USA) water purification system.

### 3.3. Sample Preparation

Berries were stored in a freezer at −80 °C until analysis. In the next step, the berries were lyophilized at the pressure of 0.01 mbar and a condenser temperature of −85 °C in a condenser. Lyophilized berries were ground using the knife mill Grindomix GM 200 (Retsch GmbH, Haan, Germany) and samples were stored in tightly closed containers until investigation.

The dehydration level of samples was determined with a hygrometer Precisa 310 M (Precisa, Dietikon, Switzerland). For each sample, the procedure was repeated three times and averages of the drying estimates were calculated.

For preparation of ethanolic extracts, each sample of 2.5 g lyophilized powder was placed in a dark glass bottle and 40 mL of 70% of ethanol at room temperature was added. Samples were sealed and placed in an ultrasonic bath and were extracted for 10 min at 80 kHz and 1017 W. After extraction, the ethanolic extracts were centrifuged for 2 min. at 8500 rpm, at room temperature using the centrifuge Heraeus Biofuge Stratos (Heraeus Holding GmbH, Haan, Germany). The supernatants were poured off from the residues, filtered and placed in dark wide-mouthed bottles, which were kept in a refrigerator at 4 °C until analysis. Ethanolic extracts were filtered through 0.22 μm pore size membrane filters prior to investigation.

### 3.4. Determination of Total Phenolic Content and Quantitative Composition by UHPLC-ESI-MS/MS Technique

All the spectrophotometric measurements were carried out with the M550 UV/Vis spectrophotometer (Spectronic CamSpec, Garforth, England, UK).

Separation of phenolic compounds was performed with the Acquity H-class UHPLC system (Waters, Santa Clara, CA, USA) equipped with a triple quadrupole tandem mass spectrometer (Xevo, Waters, USA) with an electrospray ionization source (ESI) to obtain MS/MS data using a previously described and validated UHPLC-ESI-MS/MS technique [2]. A YMC Triart C18 (100 × 2.0 mm; 1.9 μm) column (YMC Europe GmbH, Dislanken, Germany) was used for analysis. Column temperature was maintained at 40 °C. Gradient elution was performed with a mobile phase consisting of 0.1% formic acid water solution (solvent A) and acetonitrile (solvent B) with the flow rate set to 0.5 mL min^−1^. Injection volume was 10 µL. Linear gradient profile was applied as follows for solvent A: initially 95% for 1 min; to 70% over 4 min; 50% over 7 min; 95% over 2 min. Negative electrospray ionization was applied for analysis: capillary voltage −2 kV, source temperature 150 °C, desolvation temperature 400 °C, desolvation gas flow 700 L h^−1^, cone gas flow 20 L h^−1^. Each compound of interest had a specific collision energy and cone voltage selected. The selected mass spectrometry parameters for this method were presented in our previous work [2]. The evaluation of the selectivity of the method for peak identification and purity was based on the comparison of the retention times and MS spectra of the analytes with those of the standard compounds. The limit of detection (LOD) and limit of quantification (LOQ) of the analytes were assessed by comparing the peak height to the baseline noise. The signal-to-noise ratio was 3:1 for a limit of detection and it was 10:1 for a limit of quantification. The determined LOD varied from 0.4 ng/mL to 32.51 ng/mL and the determined LOQ varied from 1.2 ng/mL to 100.25 ng/mL. Calibration curves were obtained by plotting the peak areas of analytical standards. The estimated determination coefficients (R2) of calibration curves was ≥ 0.96. Content of the phenolic compound was expressed as µg/g DW. Samples of chromatograms were presented in the Supplementary Materials, Figures S1 and S2.

### 3.5. Determination of Antioxidant Activity

#### 3.5.1. DPPH• Free Radical Scavenging Assay

The DPPH• free radical scavenging activity was determined using the method proposed by Brand–Williams et al. [39,40]. DPPH• solution in 96.3% *v/v* ethanol (3 mL, 6 × 10^−5^ M) was mixed with 10 μL of the ethanol extract of *A. kolomikta* fruits or leaves. A decrease in absorbance was determined at a wavelength of 515 nm after keeping the samples for 30 min in the dark. Trolox was used as the standard for the calibration curve and the DPPH• values were expressed as µmol of trolox equivalents (µmol TE) per g DM of extract.

#### 3.5.2. CUPRAC Assay

CUPRAC solution included copper (II) chloride (0.01 M in water), ammonium acetate buffer solution (0.001 M, pH = 7), and neocuproine (0.0075 M in ethanol) (ratio 1:1:1). A volume of 3 mL of freshly prepared CUPRAC reagent was mixed with 10 μL of Actinidia fruits or leaves extract. An increase in absorbance was recorded after 30 min at a wavelength of 450 nm [30]. CUPRAC assay measurements and calculations were performed using trolox calibration curves and were expressed as µmol of the trolox equivalent (TE) per one gram of dry weight.

### 3.6. Statistical Analysis

Mean values and standard deviations were calculated using MS Excel 2020 (Redmond, WA, USA). One-way analysis of the variance (ANOVA) along with the post hoc Duncan’s test was employed for statistical analysis using SPSS Statistics version 27.0.0.0 (IBM, Armonk, NY, USA) software. Moreover, a custom chart builder was implemented using a scatter plot with a grouping variable and point ID label.

## 4. Conclusions

Investigations of *A. kolomikta* cultivars revealed a range of phenolic compounds. Both berries and leaves showed the presence of flavan-3-ols, phenolic acids, flavones, and flavonols. This indicates that *A. kolomikta* is a promising health promoting source of biochemical compounds with antioxidant activity. Berries of the cultivar ‘VIR-2′ and leaves of the cultivar ‘Aromatnaja’ accumulated the largest amounts of total phenolic compounds. There were significant differences amongst the cultivars studied but the cultivar ‘VIR-2’ as a potential donor of valuable biochemical properties in the breeding of cultivars with higher amounts of phenolic compounds in berries, was confirmed. For the first time the composition of the phenolic compounds of leaves from different of *A. kolomikta* cultivars were comprehensively evaluated, and both DPPH• and CUPRAC technologies demonstrated strong antioxidant potential. This study confirmed high levels of different classes of phenolic compounds in the leaves, thus it is important to further investigate their potential applications for the food or pharmaceutical industries.

## Figures and Tables

**Table 1:** Content of phenolic compounds (µg/g) in leaves of different *Actinidia kolomikta* cultivars (1–12).

Phenolic Compound, µg/g	Cultivar of *A. kolomikta*											
	1	2	3	4	5	6	7	8	9	10	11	12
*Flavan-3-ols*												
(−)-Epicatechin	21.81 *ef*	478.56 *b*	39.49 *e*	566.72 *a*	2.01 *f*	8.86 *f*	138.08 *d*	110.79 *c*	141.85 *d*	40.38 *e*	17.03 *ef*	151.33 *d*
(+)-Catechin	88.16 *d*	516.65 *a*	30.25 *fg*	197.61 *b*	7.71 *gh*	-	163.93 *c*	39.96 *ef*	59.59 *e*	10.87 *gh*	-	110.65 *d*
Procyanidin C1	766.59 *ef*	3888.09 *ef*	430.94 *h*	4168.91 *a*	473.16 *h*	870.36 *e*	1254.90 *d*	669.08 *fg*	1443.84 *c*	540.26 *gh*	514.95 *h*	796.95 *ef*
*Flavones*												
Acacetin	-	8.90 *a*	3.27 *c*	-	2.38 *d*	-	-	-	-	-	-	5.98 *b*
Apigenin	0.65 *c*	-	0.03 *f*	0.45 *d*	0.28 *e*	0.49 *d*	0.28 *e*	-	0.83 *b*	0.45 *d*	0.31 *e*	0.90 *a*
*Phenolic acids*												
Caffeic acid	-	-	-	8.37 *b*	-	1.94 *c*	-	-	-	-	-	9.80 *a*
Chlorogenic acid	72.24 *d*	435.17 *a*	144.10 *c*	36.5 *f*	44.33 *ef*	15.74 *g*	11.64 *g*	15.17 *g*	55.23 *e*	224.27 *b*	153.51 *c*	58.43 *de*
Neochlorogenic acid	101.37 *e*	758.44 *a*	16.87 *g*	450.52 *c*	8.64 *g*	71.12 *f*	114.24 *e*	99.36 *ef*	576.96 *b*	35.64 *g*	35.22 *g*	360.25 *d*
*Flavonols*												
Kaempherol	121.95 *e*	193.84 *d*	214.47 *cd*	460.19 *a*	220.45 *c*	190.61 *d*	35.27 *f*	135.68 *e*	373.36 b	471.56 *a*	470.34 *a*	222.81 *c*
Kaempherol-3-*O*-glucoside	344.32 *g*	902.35 *c*	439.04 *f*	1119.18 *b*	221.24 *i*	825.13 *d*	783.29 *d*	256.86 *hi*	1309.04 *a*	604.09 *e*	305.23 *gh*	550.80 *e*
Quercetin	35.80 *i*	108.77 *d*	47.96 *h*	209.17 *a*	59.65 *fg*	55.37 *fgh*	11.66 *j*	63.75 *f*	79.52 *e*	144.67 *b*	121.14 *c*	54.41 *gh*
Galangin	-	3.55 *a*	-	-	-	-	-	-	-	-	-	-
Phloridzin	-	-	-	-	-	-	-	-	-	-	1.68 *a*	-
Quercitrin	2.80 *e*	0.87 *h*	14.12 *a*	2.84 *e*	5.05 *c*	2.37 *f*	1.41 *g*	1.76 *g*	2.8 *e*	1.74 *g*	11.2 *b*	3.53 *d*
Rutin	29.87 *d*	67.52 *a*	52.58 *b*	24.72 *e*	8.97 *h*	5.50 *i*	17.52 *g*	15.72 *g*	32.66 *c*	23.88 *ef*	21.58 *f*	18.25 *g*
Isorhamnetin	291.57 *c*	317.02 *c*	168.13 *f*	311.99 *c*	366.56 *b*	348.73 *b*	96.04 *g*	254.19 *d*	207.19 *e*	351.35 *b*	432.77 *a*	298.72 *c*
Isorhamnetin-3-*O*-rutinoside	0.57 *b*	-	-	-	-	-	-	-	-	0.70 *a*	-	-

Values followed by the same letter, within the same row, are not significantly different according to Duncan’s least significant difference (LSD) procedure at 5% significance level. Cultivar numbers where: 1—‘Anykšta’; 2—‘Aromatnaja’; 3—‘Krupnoplodnaja’; 4—‘Laiba’; 5—‘Landė’; 6—‘Lankė’; 7—‘Matovaja’; 8—‘Paukstės Sakarva’; 9—‘Pavlovskaja’; 10—‘Sentiabrskaja’; 11—‘VIR-1’; 12—‘VIR-2’. Different letters in the row described significant differences between cultivars.

**Table 2:** Content of phenolic compounds (µg/g) in berries of different *Actinidia kolomikta* cultivars (1–12).

Phenolic Compound, µg/g	Cultivar of *A. kolomikta*											
	1	2	3	4	5	6	7	8	9	10	11	12
*Flavan-3-ols*												
(−)-Epicatechin	178.21 *b*	96.75 *g*	117.44 *de*	-	100.05 *fg*	125.96 *d*	106.92 *f*	124.83 *d*	147.21 *c*	109.81 *ef*	107.78 *ef*	204.44 *a*
(+)-Catechin	9.64 *f*	-	4.12 *i*	-	5.59 *h*	14.92 *de*	13.89 *e*	15.63 *cd*	16.10 *c*	17.54 *b*	6.76 *g*	27.17 *a*
Procyanidin C1	832.34 *b*	729.8 *c*	708.89 *c*	-	567.27 *d*	577.97 *d*	316.04 *f*	563.67 *d*	840.5 *b*	409.61 *e*	548.15 *d*	1236.66 *a*
*Flavones*												
Apigenin	0.07 *e*	0.05 *f*	-	-	0.22 *b*	0.33 *a*	0.02 *g*	-	0.09 *d*	0.14 *c*	-	0.09 *d*
*Phenolic acids*												
Caffeic acid	-	-	-	-	-	2.62 *a*	-	-	-	-	-	-
Chlorogenic acid	54.49 *ef*	62.37 *d*	75.71 *c*	-	23.72 *g*	21.6 *g*	14.48 *h*	88.24 *b*	52.08 *f*	84.46 *b*	125.92 *a*	58.36 *de*
Neochlorogenic acid	-	-	-	-	-	-	-	-	-	-	-	0.57 *a*
*Flavonols*												
Kaempherol	29.32 *c*	35.25 *a*	-	-	31.46 *b*	-	25.45 *d*	10.25 *f*	3.63 *g*	19.86 *e*	3.44 *g*	4.74 *g*
Kaempherol-3-*O*-glucoside	97.83 *h*	380.98 *b*	401.75 *b*	-	228.64 *e*	479.49 *a*	197.17 *f*	275.1 *d*	351.48 *c*	129.98 *g*	179.11 *f*	91.86 *h*
Quercetin	67.79 *b*	7.1 *e*	1.27 *f*	-	4.46 *e*	82.54 *a*	4.33 *e*	24.23 *c*	13.17 *d*	5.45 *e*	-	4.46 *e*
Quercitrin	1.8 *a*	1.75 *ab*	1.4 *c*	-	0.89 *e*	1.38 *c*	0.66 *f*	1.47 *c*	1.67 *b*	0.73 *f*	1.0 *e*	1.2 *d*
Rutin	24.29 *b*	7.76 *gh*	9.47 *efg*	0	7.7 *h*	9.77 *ef*	10.44 *e*	13.63 *d*	15.46 *c*	2.69 *i*	8.33 *fgh*	54.94 *a*
Isorhamnetin	20.32 *c*	42.82 *a*	-	-	15.16 *d*	15.83 *d*	31.26 *b*	-	9.74 *e*	-	4.12 *f*	-

Values followed by the same letter, within the same row, are not significantly different according to Duncan’s least significant difference (LSD) procedure at 5% significance level. Cultivar numbers where: 1—‘Anykšta’; 2—‘Aromatnaja’; 3—‘Krupnoplodnaja’; 4—‘Laiba’; 5—‘Landė’; 6—‘Lankė’; 7—‘Matovaja’; 8—‘Paukstes Sakarva’; 9—‘Pavlovskaja’; 10—‘Sentiabrskaja’; 11—‘VIR-1’; 12—‘VIR-2’. Different letters in the row described significant differences between cultivars.

**Table 3:** Summary content of phenolic compounds (µg/g) in berries and leaves of different *Actinidia kolomikta* cultivars.

Cultivar/Clone	Part of Plant	Total Flavan-3-Ols	Total Flavones	Total Phenolic Acids	Total Flavonols	∑ Phenolic Compounds
‘Anykšta’	BERRIES	1020.18 ± 23.9 *b*	0.07 ± 0 *e*	54.49 ± 0.98 *ef*	241.35 ± 8.42 *f*	1316.09 ± 33.05 *c*
‘Aromatnaja’		826.55 ± 31.77 *c*	0.05 ± 0 *f*	62.37 ± 2.12 *d*	475.67 ± 16.38 *b*	1364.64 ± 50.12 *bc*
‘Krupnoplodnaja’		830.46 ± 18.6 *c*	0 ± 0 *h*	75.71 ± 2.7 *c*	413.89 ± 8 *c*	1320.06 ± 29.25 *c*
‘Laiba’		-	-	-	-	-
‘Landė’		672.9 ± 17.97 *d*	0.22 ± 0.01 *b*	23.72 ± 0.77 *g*	288.31 ± 8.34 *e*	985.15 ± 26.97 *e*
‘Lankė’		718.85 ± 17.15 *d*	0.33 ± 0.01 *a*	24.23 ± 0.78 *g*	589.01 ± 17.49 *a*	1332.42 ± 34.95 *c*
‘Matovaja’		436.84 ± 16.62 *f*	0.02 ± 0 *g*	14.48 ± 0.44 *h*	269.31 ± 6.14 *ef*	720.64 ± 23.12 *f*
‘Paukštės Šakarva’		704.13 ± 13.87 *d*	0 ± 0 *h*	88.24 ± 2.64 *b*	324.68 ± 12.02 *d*	1117.05 ± 28.21 *d*
‘Pavlovskaja’		1003.81 ± 35.22 *b*	0.09 ± 0 *d*	52.08 ± 1.13 *f*	395.15 ± 13.05 *c*	1451.13 ± 49.11 *b*
‘Sentiabrskaja’		536.97 ± 10.09 *e*	0.14 ± 0.01 *c*	84.46 ± 3.37 *b*	158.72 ± 6.05 *h*	780.29 ± 19.49 *f*
‘VIR-1’		662.69 ± 26.95 *d*	0 ± 0 *h*	125.92 ± 2.19 *a*	196 ± 8.12 *g*	984.61 ± 37.23 *e*
‘VIR-2’		1468.26 ± 39.18 *a*	0.09 ± 0 *d*	58.92 ± 0.96 *de*	157.21 ± 3.24 *h*	1684.48 ± 43.35 *a*
‘Anykšta’	LEAVES	876.55 ± 31.77 *cd*	0.65 ± 0.02 *ef*	173.61 ± 4.51 *f*	826.87 ± 31 *gh*	1877.7 ± 67.1 *ef*
‘Aromatnaja’		4883.3 ± 131.11 *a*	8.9 ± 0.33 *a*	1193.61 ± 37.21 *a*	1593.91 ± 38.43 *c*	7679.72 ± 206.67 *a*
‘Krupnoplodnaja’		500.67 ± 16.79 *e*	3.31 ± 0.1 *c*	160.96 ± 2.86 *fg*	936.29 ± 33.58 *fg*	1601.23 ± 53.08 *fg*
‘Laiba’		4933.24 ± 164.67 *a*	0.45 ± 0.02 *fg*	495.4 ± 16.85 *c*	2128.08 ± 54.45 *a*	7557.17 ± 235.41 *a*
‘Landė’		482.89 ± 19.15 *e*	2.66 ± 0.06 *d*	52.97 ± 1.85 *i*	881.91 ± 28.8 *fg*	1420.44 ± 49.85 *g*
‘Lankė’		879.22 ± 23.82 *cd*	0.49 ± 0.02 *efg*	88.8 ± 3.81 *hi*	1427.71 ± 48.81 *d*	2396.21 ± 76.32 *cd*
‘Matovaja’		1556.9 ± 32.85 *b*	0.28 ± 0.01 *gh*	125.88 ± 4.98 *gh*	945.2 ± 32.75 *f*	2628.27 ± 70.14 *c*
‘Paukštės Šakarva’		819.82 ± 19.66 *d*	0 ± 0 *h*	114.53 ± 3.38 *h*	727.96 ± 18.55 *h*	1662.32 ± 41.48 *fg*
‘Pavlovskaja’		1645.27 ± 38.97 *b*	0.83 ± 0.02 *e*	632.18 ± 15.91 *b*	2004.58 ± 52.9 *b*	4282.87 ± 107.28 *b*
‘Sentiabrskaja’		591.51 ± 8.96 *e*	0.45 ± 0.01 *fg*	259.92 ± 9.19 *e*	1597.99 ± 46.32 *c*	2449.86 ± 64.41 *c*
‘VIR-1’		531.98 ± 17.86 *e*	0.31 ± 0.01 *fgh*	188.73 ± 7.24 *f*	1363.94 ± 31.73 *d*	2084.96 ± 56.75 *de*
‘VIR-2’		1058.93 ± 26.95 *c*	6.88 ± 0.27 *b*	428.47 ± 14.16 *d*	1148.53 ± 43.84 *e*	2642.81 ± 85.04 *c*

Values followed by the same letter, within the same column, are not significantly different according to Duncan’s least significant difference (LSD) procedure at 5% significance level.

**Table 4:** List of *Actinidia kolomikta* cultivars investigated in the present study.

	Cultivar	Origin	Berries Analyses	Leaves Analyses
1	‘Anykšta’	Private collection, Lithuania	+	+
2	‘Aromatnaja’	Pavlovsk Research Station, Russian Federation	+	+
3	‘Krupnoplodnaja’	Pavlovsk Research Station, Russian Federation	+	+
4	‘Laiba’	VMU Agriculture Academy, Lithuania	−	+
5	‘Landė’	VMU Agriculture Academy, Lithuania	+	+
6	‘Lankė’	VMU Agriculture Academy, Lithuania	+	+
7	‘Matovaja’	Pavlovsk Research Station, Russian Federation	+	+
8	‘Paukstės Sakarva’	VMU Agriculture Academy, Lithuania	+	+
9	‘Pavlovskaja’	Pavlovsk Research Station, Russian Federation	+	+
10	‘Sentiabrskaja’	Pavlovsk Research Station, Russian Federation	+	+
11	‘VIR-1’	Pavlovsk Research Station, Russian Federation	+	+
12	‘VIR-2’	Pavlovsk Research Station, Russian Federation	+	+

## Data Availability

The data presented in this study are available in the article.

## Supplementary Materials

The following supporting information can be downloaded at: https://www.mdpi.com/article/10.3390/plants11020147/s1, Figure S1: Chromatogram of leaves of the cultivar ‘Landė’; Figure S2: Chromatogram of berries of the cultivar ‘Landė’.
