# Supplementary figures and images for: Berries and Leaves of Actinidia kolomikta (Rupr. & Maxim.) Maxim.: A Source of Phenolic Compounds
Source: Plants (Basel). 2022 Jan 6;11(2):147. doi: 10.3390/plants11020147 (PMC8781454; doi:10.3390/plants11020147)

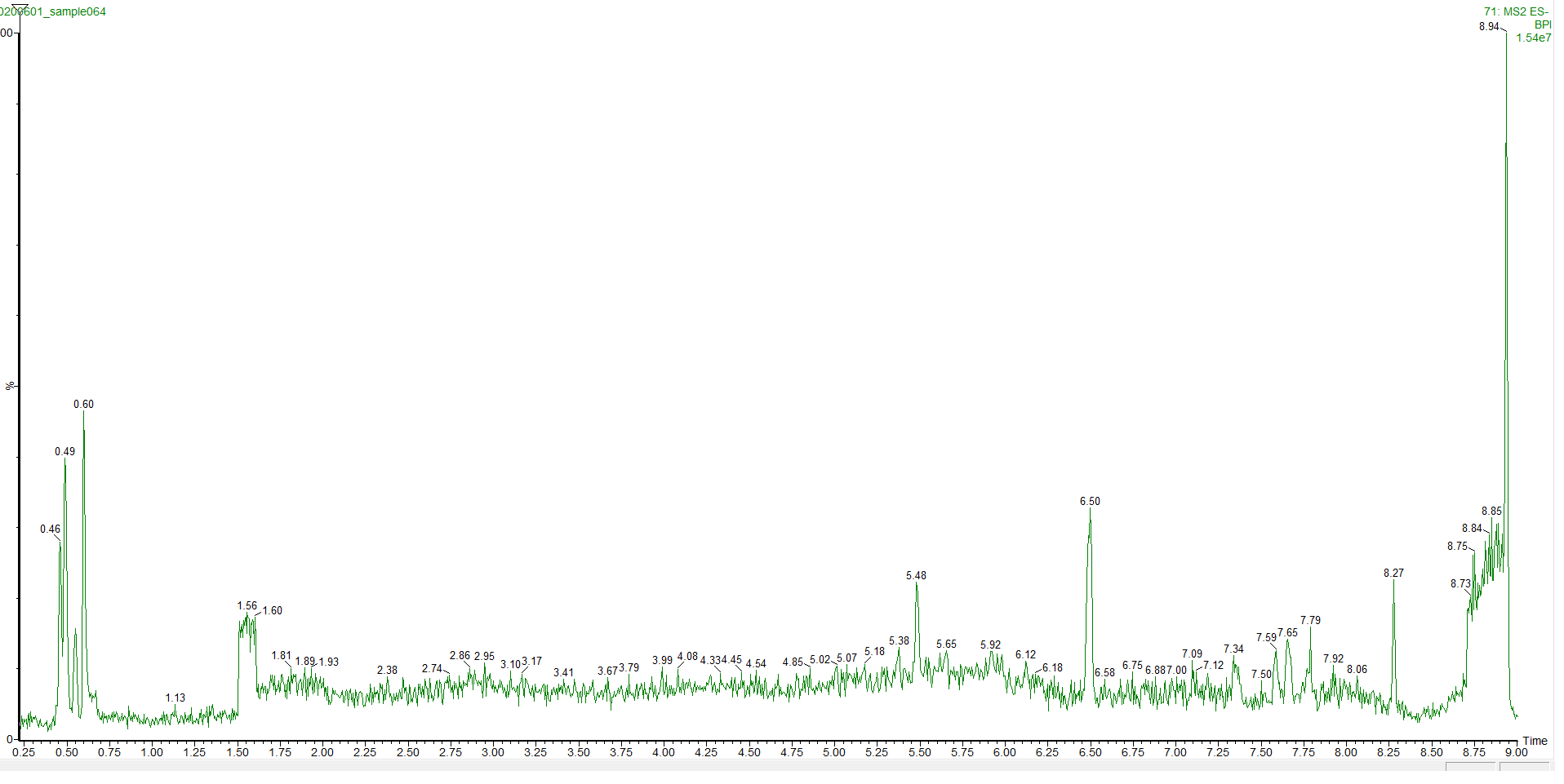

Supplement: Supplementary file 1 [file plants-11-00147-s001.zip › Figure S1.tif]

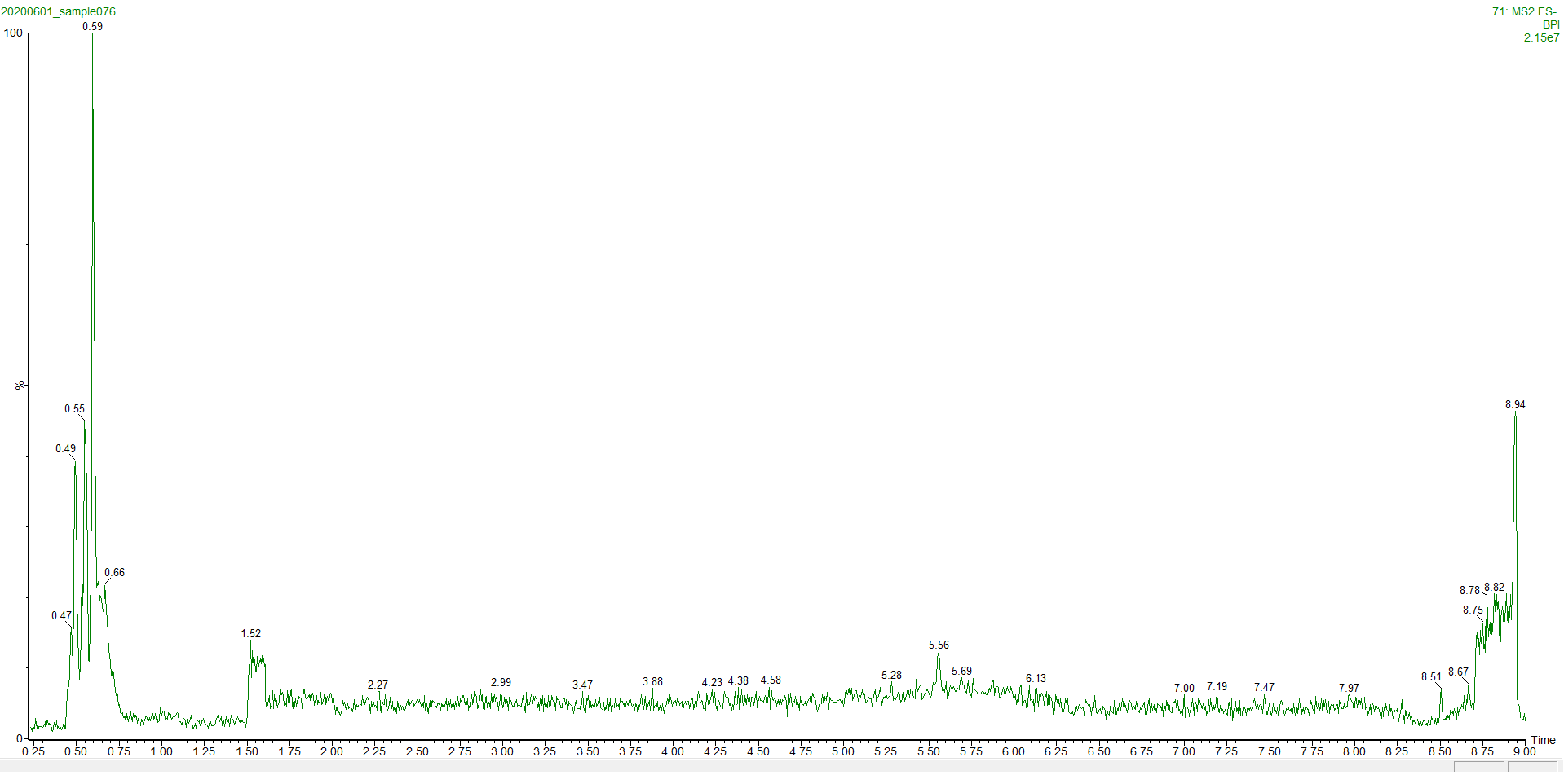

Supplement: Supplementary file 1 [file plants-11-00147-s001.zip › Figure S2.tif]
